# Novel regulatory mechanism of serine biosynthesis associated with 3-phosphoglycerate dehydrogenase in *Arabidopsis thaliana*

**DOI:** 10.1038/s41598-017-03807-5

**Published:** 2017-06-14

**Authors:** Eiji Okamura, Masami Yokota Hirai

**Affiliations:** RIKEN Center for Sustainable Resource Science, 1-7-22 Suehiro-cho, Tsurumi-ku, Yokohama, Kanagawa 230-0045 Japan

## Abstract

The proteinogenic amino acid l-serine is a precursor for various essential biomolecules in all organisms. 3-Phosphoglycerate dehydrogenase (PGDH) is the first committed enzyme of the phosphorylated pathway of l-serine biosynthesis, and is regulated by negative feedback from l-serine in bacteria and plants. In the present study, two Arabidopsis PGDH isoforms were inhibited by l-serine but were activated by l-amino acids such as l-homocysteine *in vitro*. Activation and inhibition by these amino acids was cooperative, suggesting an allosteric mechanism. Moreover, the half maximal effective concentration of l-homocysteine was 2 orders of magnitude lower than that of l-serine, suggesting greater regulatory potency. These are the first data to show that PGDH is activated by various biomolecules and indicate that serine biosynthesis is regulated by multiple pathways.

## Introduction

**Figure 1:**
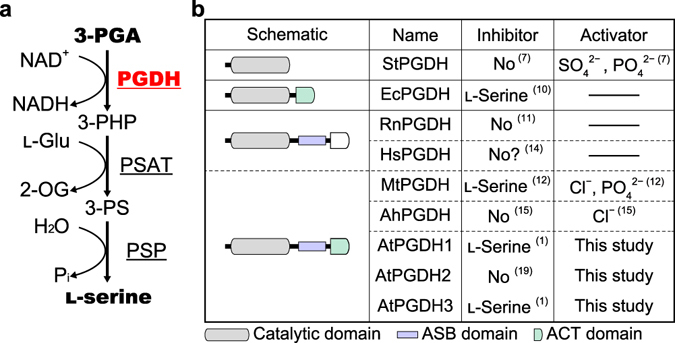
Enzymatic functions, domain architectures, and effectors of 3-phosphoglycerate dehydrogenase. (**a**) Schematic of the phosphorylated pathway; names of enzymes are underlined; PGDH, 3-phosphoglycerate dehydrogenase; PSAT, phosphoserine aminotransferase; PSP, phosphoserine phosphatase; 3-PHP, 3-phosphohydroxypyruvate; 3-PS, 3-phosphoserine; l-Glu, l-glutamate; 2-OG, 2-oxoglutarate; Pi, inorganic phosphate. (**b**) Domain architectures and effectors of PGDH; the domains of PGDH proteins from archaea [*S*. *tokodaii* (StPGDH)], bacteria [*E*. *coli* (EcPGDH) and *M*. *tuberculosis* (MtPGDH)], cyanobacterium [*A*. *halophytica* (AhPGDH)], mammals [*R*. *norvegicus* (RnPGDH) and *Homo sapiens* (HsPGDH)], and plant [*A*. *thaliana* (AtPGDHs)] are schematically shown with highlighted catalytic, ACT, and ASB domains. Because ACT domains of HsPGDH and RnPGDH are not functional, they are indicated in white. Numbers in parenthesis indicate references. In ref. 14, HsPGDH was not inhibited by serine, although the data were not shown (“No ? ” in the figure).

In plants, l-serine is predominantly synthesized by glycolate and phosphorylated pathways. The glycolate pathway occurs in the mitochondria and is part of the photorespiratory pathway. This route is considered the main biosynthetic pathway of l-serine, at least in photosynthetic cells^1, 2^. The phosphorylated pathway (Fig. 1a), which is found in mammals, bacteria, and plants, comprises reactions of 3-phosphoglycerate dehydrogenase (PGDH), phosphoserine aminotransferase, and phosphoserine phosphatase^2^, and occurs in plastids^3^. The first committed enzyme PGDH oxidizes 3-phosphoglycerate (3-PGA), which originates from glycolysis and the Calvin cycle in heterotrophic and photosynthetic autotroph cells^2, 4, 5^.

PGDH isoforms are classified according to protein domain structures^6^, and whereas PGDHs from organisms such as *Sulfolobus tokodaii* comprise only the catalytic domain^7^, other PGDHs possess an aspartate kinase–chorismate mutase–TyrA (ACT) domain and, in some cases, an allosteric substrate binding (ASB) domain (Fig. 1b)^6^. PGDH from *Escherichia coli* (EcPGDH) possesses only an additional ACT domain, and is allosterically and cooperatively inhibited by l-serine to achieve negative feedback regulation of the phosphorylated pathway^8^. Binding of l-serine to the ACT domain changes the conformation of EcPGDH and lowers its catalytic rate^9, 10^. However, PGDH from *Rattus norvegicus* (RnPGDH) is considered to possess both ACT and ASB domains in addition to the catalytic domain, but is not inhibited by l-serine^6, 11^, likely due to the reported absence of critical amino acid residues in the ACT domain^6^. PGDH from *Mycobacterium tuberculosis* (MtPGDH) comprises ACT, ASB, and catalytic domains and is cooperatively inhibited by l-serine in the presence of anions such as phosphate and chloride, and is non-cooperatively inhibited in the absence of these anions^12, 13^. Depending on phosphate concentrations, MtPGDH forms homodimers, homotetramers, and homooctamers that vary in catalytic activity and sensitivity to inhibition by l-serine^13^. In active and l-serine-sensitive homotetramers and homooctamers, phosphate binds to the pocket formed by ASB and ACT domains of two PGDH molecules, and l-serine binds the ACT domain interface as previously reported^6, 13, 14^. In contrast, PGDH from *Aphanothece halophytica* (AhPGDH) is not inhibited by l-serine, despite the presence of the putative ACT domain^15^. This indicates that the mechanism of allosteric inhibition by l-serine is not solely attributed to the presence of the ACT domain.

*Arabidopsis thaliana* expresses three PGDH isoforms and these are encoded by *PGDH1* (At4g34200), *PGDH2* (At1g17745), and *PGDH3* (At3g19480)^1^ (henceforth designated *AtPGDH1*, *AtPGDH2*, and *AtPGDH3*, respectively). Although AtPGDH1 and AtPGDH3, but not AtPGDH2, are inhibited by l-serine^1^, the related modes of inhibition remain unknown. In plants, l-serine provides the carbon skeleton for the synthesis of l-cysteine and l-tryptophan, which are further converted into important biomolecules such as *S*-adenosyl-l-methionine (AdoMet) and the phytohormone indole acetic acid, respectively^16, 17^. Therefore, we hypothesized that PGDH is regulated by multiple amino acids, as shown for aspartate kinase in the aspartate-family amino acid biosynthesis^18^, and determined the effects of various amino acids on AtPGDH activity in the presence and absence of l-serine.

In the present study, AtPGDH1 and AtPGDH3 were cooperatively activated by l-aspartate-﻿﻿derived l-homocysteine, l-methionine, and l-homoserine, by pyruvate﻿-﻿﻿derived l-alanine and l-valine, and by l-serine-﻿﻿derived l-cysteine. Among these activators of AtPGDH, l-homocysteine had the lowest half maximal effective concentration (EC_50_). The results suggested that the supply of l-serine is more refined than our conventional understanding in balancing the whole metabolic network.

## Domain structures and biochemical properties of AtPGDHs

**Figure 2:**
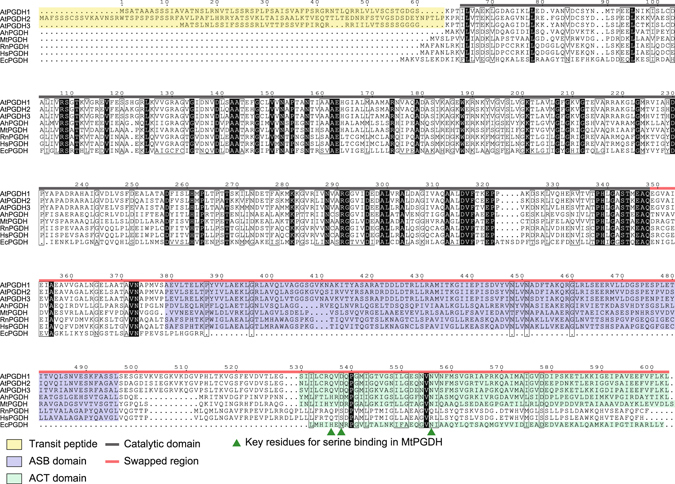
Multiple sequence alignments of PGDHs. Amino acid sequences of PGDHs from *A*. *thaliana* (AtPGDH), *A*. *halophytica* (AhPGDH), *M*. *tuberculosis* (MtPGDH), *R*. *norvegicus* (RnPGDH), *H*. *sapiens* (HsPGDH), and *E*. *coli* (EcPGDH) are shown with predicted transit peptides (yellow), ASB domains (violet), and ACT domains (green). Black and red lines indicate catalytic domains and swapped regions in domain swapping experiments, respectively. Green triangles represent the key l-serine binding residues in MtPGDH.

Multiple sequence alignments of AtPGDH1, AtPGDH2, and AtPGDH3 were performed with PGDHs from bacteria (EcPGDH, MtPGDH, and AhPGDH) and mammals (HsPGDH from *Homo sapiens* and RnPGDH from *Rattus norvegicus*; Fig. 2). All three Arabidopsis isoforms were previously shown to carry putative transit peptides for chloroplast localization^1, 19, 20^, and the catalytic domain for oxidation of 3-PGA and concomitant reduction of NAD^+^ is located in N-terminal regions of all enzymes examined. Amino acid sequences of the catalytic domains of AtPGDHs have 42.2–44.8% identity with those of MtPGDH, whereas the C-terminal regions of PGDHs have low homology. However, all PGDHs except EcPGDH were predicted by InterPro^21^ to possess the ASB domain, and AtPGDHs were predicted to have ACT domains that are similar to AhPGDH, MtPGDH, and EcPGDH. The amino acid sequences of ASB and ACT domains of AtPGDHs had 15.6–17.9% and 19.1–21.9% identity to those of MtPGDH, respectively.

**Figure 3:**
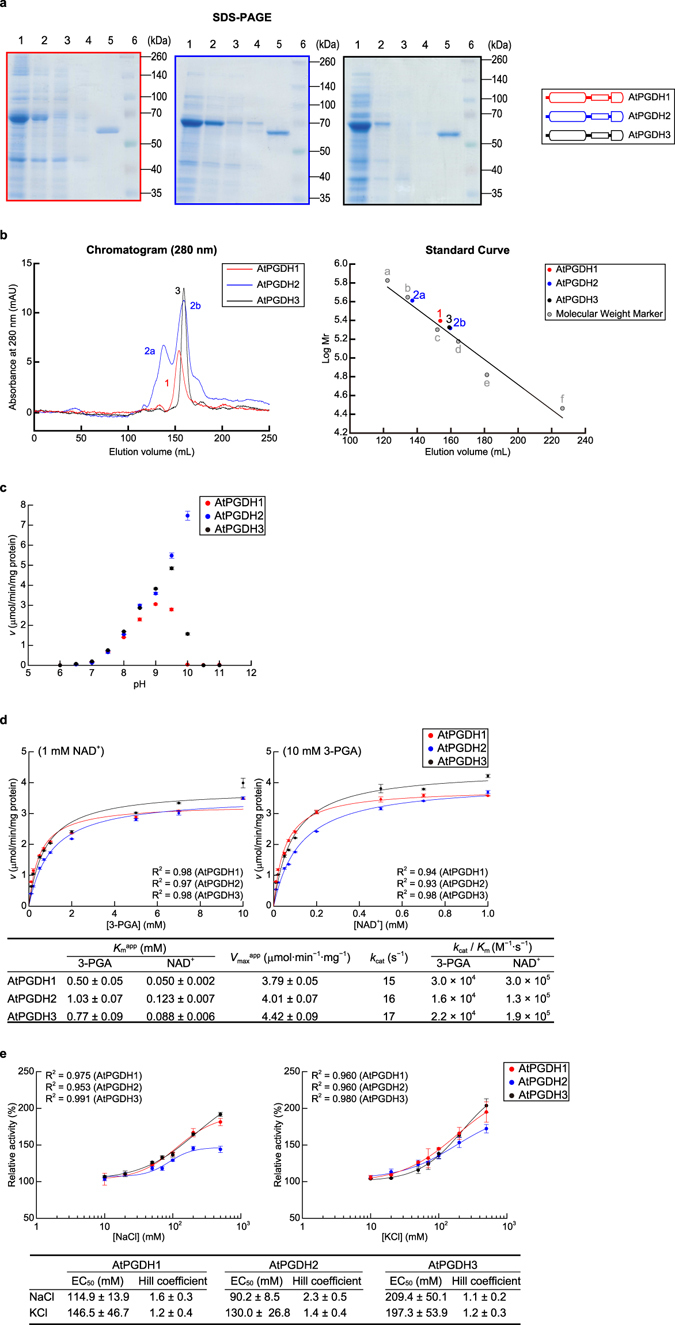
Characteristics of AtPGDHs. (**a**) SDS-PAGE analysis of recombinant AtPGDHs; 1, total protein; 2, supernatant; 3, flow through; 4, washed fraction; 5, elution fraction; 6, molecular weight marker. (**b**) Size exclusion chromatography analysis of recombinant AtPGDHs. Left panel shows the chromatograms from size exclusion chromatography analyses. Right panel shows the standard curves for estimations of molecular weights. (**c**) Optimal pH for 3-PGA oxidation activity; specific activities of AtPGDH1, AtPGDH2, and AtPGDH3 are shown at various pH values. (**d**) Kinetic parameters for 3-PGA oxidation activity; Michaelis–Menten plots for calculating apparent Michaelis constants (*K*_m_^app^) and apparent maximum velocities (*V*_max_^app^) are shown for 3-PGA (left) and NAD^+^ (right). Data are presented as means and standard errors of three technical replicates. (**e**) Effects of chloride on 3-PGA oxidation activity; specific activities of AtPGDH isoforms at different concentrations of sodium chloride (left) or potassium chloride (right) were measured and are presented relative to the specific activity at 0 mM chloride. Data are presented as means and standard errors of two biological replicates and three technical replicates (n = 6). Half maximal effective concentrations (EC_50_) and Hill coefficients are summarized in the table.

In this study, we expressed *AtPGDH1*, *AtPGDH2*, and *AtPGDH3* genes heterologously in *E*. *coli* and analyzed the ensuing recombinant proteins using sodium dodecyl sulfate-polyacryamide gel electrophoresis (SDS-PAGE). These analyses indicated that all recombinant proteins were approximately 60 kDa, which was consistent with the theoretical molecular weights of AtPGDH1 (57.5 kDa), AtPGDH2 (57.6 kDa), and AtPGDH3 (57.9 kDa; Fig. 3a). Subsequent size exclusion chromatography analyses indicated that AtPGDH1 and AtPGDH3 predominantly form homotetramers, whereas AtPGDH2 formed an equilibrium of homooctamers and homotetramers under the present experimental conditions (Fig. 3b).

In the presence of 3-PGA as a substrate for l-serine synthesis, optimal pH values for the forward reaction were 9.0 (AtPGDH1), 10.0 (AtPGDH2), and 9.5 (AtPGDH3), respectively (Fig. 3c). This result is corroborated by the previous report stating that the optimal pH of PGDH purified from soybean root nodules was approximately 9.5^22^. Although AtPGDHs are localized to plastids^1^, in which pH was shown to be 7.2^23^, we conducted further biochemical experiments at pH 9.0. We determined apparent Michaelis constants (*K*_m_^app^) and apparent maximum velocities (*V*_max_^app^) of AtPGDH isoforms for 3-PGA and NAD^+^ (Fig. 3d). These values were similar among AtPGDH isoforms, indicating similar catalytic efficiencies of these isoforms at pH 9.0.

Some PGDHs, including MtPGDH, AhPGDH, and those from soybean root nodules, were activated by anions such as chloride in multiple previous studies^12, 15, 22^, and all of the present AtPGDH isoforms were activated by chloride ions (Fig. 3e). To determine whether activation by chloride ions is cooperative, we plotted relative activities at various concentrations of chloride ions and fitted a sigmoidal E_max_ model with baseline (Hill equation)^24, 25^ to calculate Hill coefficients (n_H_). In these computations, n_H_ = 1 indicates no cooperativity, and as summarized in Fig. 3e, Hill coefficients for activation were relatively small except for that for activation of AtPGDH2 by sodium chloride (2.3 ± 0.5), suggesting cooperativity.

## 2-Oxoglutarate is not a good substrate of AtPGDHs

**Figure 4:**
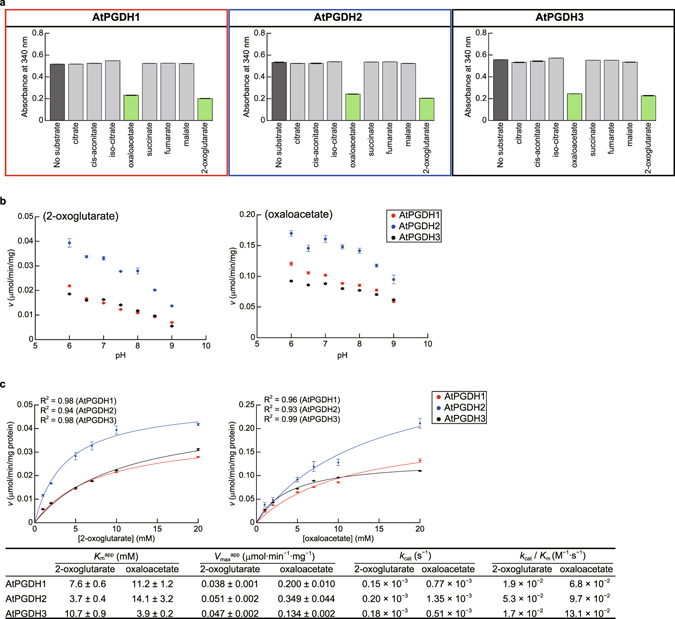
Carboxylic acid reduction activities of AtPGDHs. (**a**) Determination of substrate specificity at pH 9.0; catalytic reductions of carboxylic acids of the tricarboxylic acid (TCA) cycle were tested by monitoring decreases in absorbance of the reaction mixture at 340 nm during concomitant oxidation of the cofactor NADH for 12-h in the presence of possible substrates. Green bars indicate statistically significant reductions of in absorbance (*p* < 0.001) compared with the control (no substrate). (**b**) Optimal pH values for the reduction of 2-oxoglutarate (left) and oxaloacetate (right); specific activities of AtPGDH1, AtPGDH2, and AtPGDH3 at various pH values are shown. (**c**) Kinetic parameters for 2-oxoglutarate and oxaloacetate reduction activity; Michaelis–Menten plots for calculating apparent Michaelis constants (*K*_m_^app^) and apparent maximum velocities (*V*_max_^app^) are shown for 2-oxoglutarate (left) and oxaloacetate (right). Data are presented as means and standard errors of three technical replicates.

Because PGDHs from *E*. *coli* and human reduce 2-oxoglutarate^26, 27^ ﻿and human PGDH also reduces oxaloacetate^27^, we determined the reducing activities of the three AtPGDH isoforms with the substrates 2-oxoglutarate, oxaloacetate, and various carboxylic acids of the tricarboxylic acid (TCA) cycle (Fig. 4a). These analyses showed that only 2-oxoglutarate and oxaloacetate are substrates of AtPGDHs, and the optimal pH for reduction of 2-oxoglutarate and oxaloacetate was 6.0 (Fig. 4b). All AtPGDH isoforms showed Michaelis–Menten type curve at pH 6.0, with *K*_m_^app^ values that were 3–22 times higher than for 3-PGA (Fig. 4c). However, specificity constants *k*_cat_/*K*_m_ of AtPGDH isoforms for 2-oxoglutarate and oxaloacetate were 6 orders of magnitude lower than those for 3-PGA, indicating that the three AtPGDH isoforms specifically oxidize 3-PGA for serine biosynthesis.

## Novel cooperative activators of AtPGDHs

**Figure 5:**
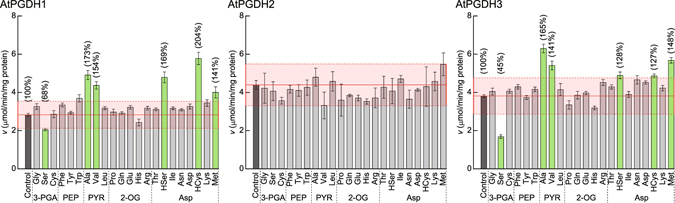
Effects of l-amino acids on 3-PGA oxidation activities of AtPGDHs. Vertical axes represent specific activities. Green bars indicate statistically significant differences (*p* < 0.001) with >25% changes compared with the specific activities in the absence of amino acids (control). The red shaded area indicates changes of <25% compared with the control. Percentages above the green bars indicate activities relative to the control. Data are presented as means and standard errors of two biological replicates and three technical replicates (n = 6). The names of precursors of effector amino acids are shown below the names of amino acids; 3-PGA, 3-phosphoglycerate; PEP, phosphoenolpyruvate; PYR, pyruvate; 2-OG, 2-oxoglutarate; and Asp, aspartate.

In previous studies, AtPGDH1 and AtPGDH3 activities were inhibited by serine^1^, whereas AtPGDH2 activity was not affected by serine, threonine, valine, glycine, tryptophan, *O*-acetyl-l-serine, or cysteine^1, 19^. In this study, we determined the effects of a total of 43 amino acids on AtPGDH activity at 10 mM (Fig. 5, Supplementary Fig. 1). Consistent with previous reports, l-serine inhibited AtPGDH1 and AtPGDH3 by 32% and 55%, respectively, but did not inhibit AtPGDH2. Moreover, l-alanine and l-valine, which are derived from pyruvate, significantly activated AtPGDH1 and AtPGDH3 (p < 0.001) by 140–170%. l-homoserine, l-homocysteine and l-methionine, which are derived from l-aspartate, also significantly activated these isoforms by 130–200%. However, d-form enantiomers of these amino acids did not activate AtPGDHs (Supplementary Fig. 1), indicating that these observations have physiological relevance. In contrast with AtPGDH1 and AtPGDH3, none of the present amino acids affected AtPGDH2 activity by more than 25% (p < 0.001). The sulfur-containing amino acids l-homocysteine and l-methionine activated AtPGDH1 and AtPGDH3. Because the sulfur atoms of these amino acids are derived from l-cysteine and the structures of l-serine and l-homocysteine are similar to that of l-cysteine, we performed further analyses to determine whether l-cysteine affects AtPGDH activity at higher concentrations.

**Figure 6:**
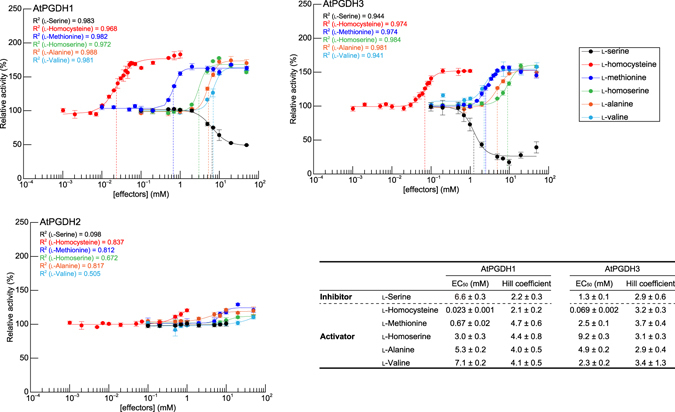
Dose responses of AtPGDH 3-PGA-oxidation activities to effector amino acids. Specific activities of AtPGDHs at various concentrations of effectors were measured and are expressed relative to those in the absence of effectors. Data are presented as means and standard errors of six technical replicates (n = 6). Regression curves of Hill equations are shown with determination coefficients (R^2^). Half maximal effective concentrations (EC_50_) and Hill coefficients of effector amino acids are summarized in the table.

To investigate modes of inhibition and activation by effector amino acids, relative activities of AtPGDHs were measured at various concentrations of effector amino acids and l-cysteine. Non-linear regression analyses of sigmoidal response curves of AtPGDH1 and AtPGDH3 revealed that absolute Hill coefficient values for l-serine, l-homocysteine, l-methionine, l-homoserine, l-alanine, and l-valine were greater than 2.0, indicating that these amino acids regulate AtPGDH activity in a cooperative manner (Fig. 6). Accordingly, whereas l- and d-cysteine were inhibitory, l-cysteine activated AtPGDH1 and AtPGDH3 at higher concentrations (Supplementary Fig. 2). Moreover, EC_50_ values of the inhibitor l-serine for AtPGDH1 and AtPGDH3 were 6.6 and 1.3 mM, respectively, as shown previously^1^. These values were much higher than those of l-serine for MtPGDH (approximately 30 µM in the presence of 150 mM NaCl)^12^ and EcPGDH (approximately 8 µM)^10^, but were within the biological range of l-serine concentrations in chloroplasts (1 to >10 mM)^28, 29^. Among activators of AtPGDH1 and AtPGDH3, l-homocysteine had the lowest EC_50_ values, at 23 µM for AtPGDH1 and 69 µM for AtPGDH3. In contrast, EC_50_ values of l-homoserine, l-alanine, and l-valine for AtPGDH1 and AtPGDH3 were in the mM order, and l-methionine affected AtPGDH1 activity at 0.67 mM. These data indicate that among effector amino acids, l-homocysteine is the most potent. Previously, l-serine, l-homoserine, l-alanine, l-valine, and l-methionine were present at 1.32, 1.52, 9.08, 1.36, and 0.12 mM, respectively, in chloroplasts from pea leaves^29^. In addition, ratios of pool sizes of methionine and homocysteine in *Chlorella sorokiniana* indicated that homocysteine was present at 0.47 mM^30^. Accordingly, the present EC_50_ values for AtPGDH1 and AtPGDH3 are within the biological ranges of these amino acid concentrations.

## Domains required for regulation by effector amino acids

**Figure 7:**
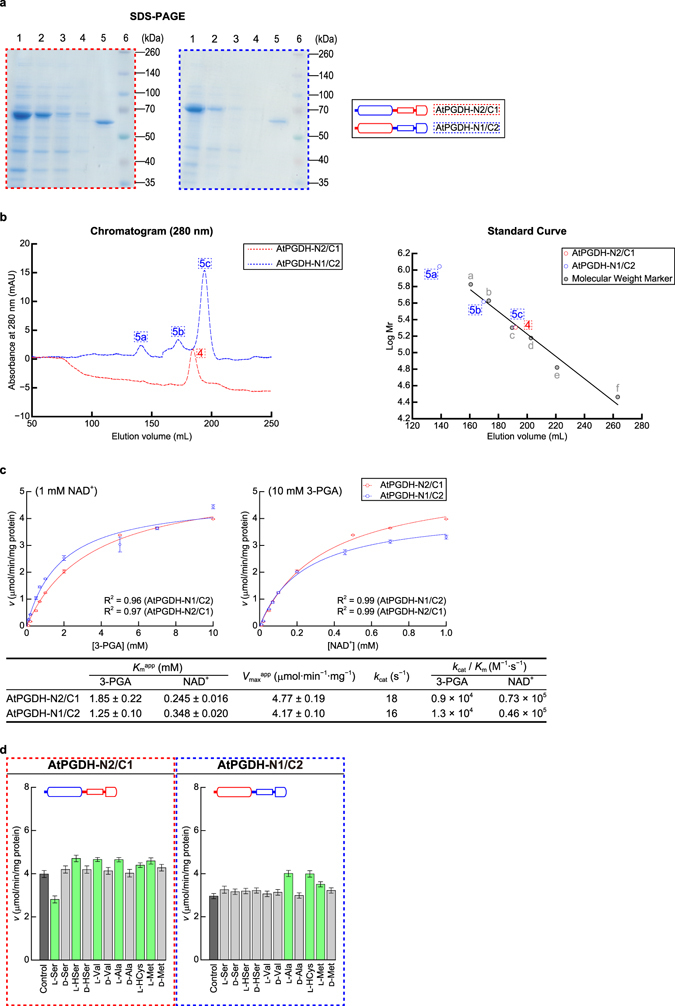
Characteristics of chimeric AtPGDHs. (**a**) SDS-PAGE analysis of recombinant AtPGDHs; 1, total protein; 2, supernatant; 3, flow through; 4, washed fraction; 5, elution fraction; 6, molecular weight marker. (**b**) Size exclusion chromatography analysis of recombinant AtPGDHs. Left panel shows the chromatogram from size exclusion chromatography analysis. Right panel shows the standard curve for estimation of molecular weights. (**c**) Kinetic parameters for 3-PGA oxidation activity; Michaelis–Menten plots for calculating apparent Michaelis constants (*K*_m_^app^) and apparent maximum velocities (*V*_max_^app^) for 3-PGA (left) and NAD^+^ (right); data are presented as means and standard errors of three technical replicates. (**d**) Effects of amino acids on 3-PGA oxidation activity; vertical axes represent specific activities. Green bars indicate significant differences (*p* < 0.05) compared with specific activities in the absence of amino acids (control). Data are presented as means and standard errors of three biological replicates and three technical replicates (n = 9).

The ACT domain is considered as the amino acid-binding site in enzymes that are regulated by amino acids^31^. Thus, to confirm the site of amino acid binding, we swapped ACT domain of amino acid-sensitive AtPGDH1 with that of the insensitive AtPGDH2. In these experiments, the N-terminal half (containing the catalytic domain) of AtPGDH2 (designated as N2) was fused to the C-terminal half (containing ASB and ACT domains) of AtPGDH1 (designated as C1), and *vice versa* to construct AtPGDH-N2/C1 and AtPGDH-N1/C2 (Fig. 7a). Subsequent size exclusion chromatography analyses suggested that AtPGDH-N2/C1 forms homotetramers (Fig. 7b), whereas AtPGDH-N1/C2 was mainly found as a homotetramer, and as multimers of to up to 20 subunits. The analyses of wild-type and chimeric enzymes indicate that the C-terminal half of AtPGDH2 is involved in multimerization beyond tetramers. Kinetic parameters of these chimeric enzymes were comparable with those of wild-type enzymes, with *k*_cat_/*K*_m_ (M^−1^s^−1^) values of 0.9 × 10^4^ (3-PGA) and 0.73 × 10^5^ (NAD^+^), and 1.3 × 10^4^ (3-PGA) and 0.46 × 10^5^ (NAD^+^) for AtPGDH-N2/C1 and AtPGDH-N1/C2, respectively (Fig. 7c), and contrasting values of 1.6 × 10^4^ (3-PGA) and 1.3 × 10^5^ (NAD^+^) and 3.0 × 10^4^ (3-PGA) and 3.0 × 10^5^ (NAD^+^) for AtPGDH2 and AtPGDH1, respectively (Fig. 3). These results indicate that specific activities of these catalytic domains are not remarkably affected in these constructs.

**Figure 8:**
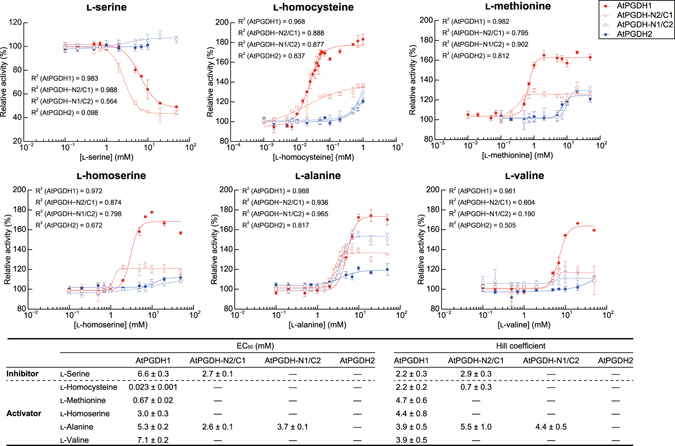
Dose responses of chimeric AtPGDH 3-PGA-oxidation activities to effector amino acids. Specific activities of chimeric enzymes at various concentrations of effector amino acids are presented relative to those measured in the absence of effectors. Data are presented as means and standard errors of three technical replicates (n = 3). For comparison, activities of AtPGDH1 and AtPGDH2 from Fig. 6 are indicated. Half maximal effective concentrations (EC_50_) for effector amino acids and Hill coefficients are summarized in the table.

We determined changes in specific activities of the chimeric enzymes following treatments with effector amino acids (Fig. 8). In these experiments, AtPGDH-N2/C1, which carries ASB and ACT domains from the amino acid-sensitive AtPGDH1, was cooperatively inhibited by l-serine, with a similar EC_50_ value to that of AtPGDH1. However, AtPGDH-N1/C2, which carries ASB and ACT domains from the amino acid-insensitive AtPGDH2, was not inhibited by l-serine. Considering the likely function of the ACT domain as an amino acid-binding site, these results suggest that l-serine binds the ACT domain of AtPGDH1, and that the C-terminal region is involved in cooperativity.

Other effector amino acids significantly activated AtPGDH-N2/C1, but with lower potency than with AtPGDH1 (Figs 7d and 8), suggesting that l-homocysteine, l-methionine, l-homoserine, and l-valine bind the C1 region. However, some features of the N1 region were required for full activation by these amino acids. l-alanine cooperatively activated both AtPGDH-N2/C1 and AtPGDH-N1/C2, with EC_50_ values that were similar to those of AtPGDH1. The data suggests that either N1 or C1 regions are sufficient for cooperative activation of AtPGDH1 by l-alanine. Moreover, consistent with the other activators, both N1 and C1 regions were necessary for full activation. In summary, these results suggest that cooperative activation mechanisms by effector amino acids require the formation of higher order structures of C- and N-terminal half regions via intra- and/or inter-molecular interactions. Moreover, this mechanism may reflect the formation of tetramers from enzymes with the C1 region, compared with the formation of multimers of proteins with the C2 region (Figs 3 and 7).

## Combined effects of l-serine and amino acid activators

**Figure 9:**
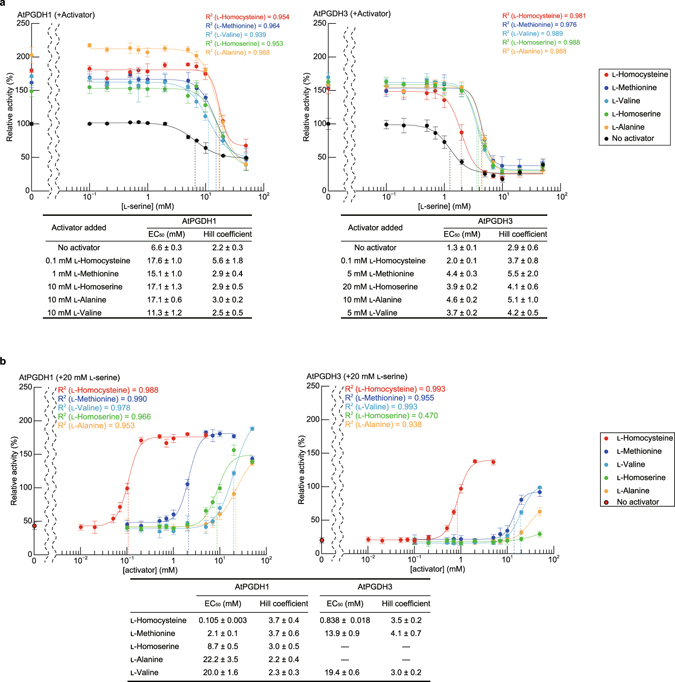
Combined effects of l-serine and activator amino acids. (**a**) Specific activities at various concentrations of l-serine were measured in the presence of each activator and are shown relative to those in the absence of l-serine. Half maximal effective concentrations (EC_50_) of l-serine and Hill coefficients are summarized in tables. (**b**) Specific activities at various concentrations of activator amino acids in the presence of 20 mM l-serine are presented relative to those in the absence of activators. Data are presented as means and standard errors of three technical replicates (n = 3). Half maximal effective concentrations (EC_50_) of effector amino acids and Hill coefficients are summarized in the table.

Because l-serine and the present activator amino acids coexist *in vivo*, we compared dose responses to l-serine in the presence of various activators *in vitro*. The EC_50_ value of l-serine for AtPGDH1 was shifted to higher concentrations in the presence of activators, although maximum inhibitory effects of l-serine were relatively unchanged (Fig. 9a). In contrast, the effect of 20 mM l-serine was decreased with increasing activator concentrations (Fig. 9b), and l-homocysteine almost completely abolished inhibition of AtPGDH1 by l-serine at concentrations of greater than 0.1 mM. Moreover, the other activators prevented inhibition of AtPGDH1 by l-serine to varying degrees, and the effects of l-homocysteine were greatest, followed by those of l-methionine. As shown for AtPGDH3, l-methionine, l-valine, l-alanine, and l-homoserine did not fully abolish inhibition by l-serine at the present activator concentrations.

## Discussion

The present chimeric enzyme experiments suggest that l-serine binds the ACT domain of AtPGDH1, and that the C-terminal region mediates cooperativity of inhibition by l-serine. MtPGDH possesses the same domain architecture as AtPGDHs and is sensitive to inhibition by l-serine, and Y461, D463, and N481 residues of the ACT domain were shown to be central to l-serine binding^14^. In AtPGDHs, the residues that correspond to D463 and N481 are conserved regardless of sensitivity to l-serine. These residues are D538 and N556 in AtPGDH1, D559 and N577 in AtPGDH2, and D523 and N541 in AtPGDH3 (Fig. 2). Although Y461 is not conserved in AtPGDHs, the corresponding residue is Q in all three isoforms. Taken together, these observations suggest that other factors play roles in differences in l-serine sensitivity between AtPGDH2 and the other two isoforms. Crystal structure analyses^32^ show that homotetramers of MtPGDH are asymmetric, reflecting the presence of the intervening domain that corresponds to the ASB domain. Moreover, the formation of one of two conformations of the regulatory domain (corresponding to the ACT domain) on each subunit likely leads to differing accessibility of l-serine. In agreement, whereas AtPGDH2 was present in an equilibrium of homotetramers and homooctamers, AtPGDH1 and AtPGDH3 only formed homotetramers (Fig. 3). Recently, a novel allosteric binding Site I was identified in EcPGDH^33^, and an ASB-homologous domain in serine dehydratase was suggested to bind the substrate serine^34^. Thus, further structural analysis of AtPGDHs may lead to the discovery of higher structures that are responsible for allosteric regulation of PGDHs.

**Figure 10:**
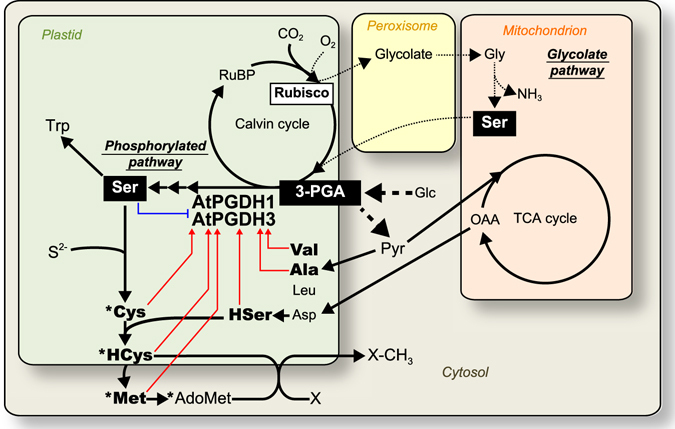
Schematic of metabolic pathways in *A*. *thaliana* focusing on the regulation of AtPGDH1 and AtPGDH3. Amino acids are denoted by standard 3-letter codes; Glc, glucose; Pyr, pyruvate; OAA, oxaloacetate; RuBP, ribulose 1,5-bisphosphate; HSer, homoserine; HCys, homocysteine; AdoMet, *S*-adenosyl-l-methionine; X, methyl acceptor; Rubisco, ribulose 1,5-bisphosphate carboxylase/oxygenase. Dashed and dotted lines indicate glycolysis and photorespiration, respectively. Red arrows and blue lines indicate activation and inhibition of AtPGDH1 and AtPGDH3, respectively. Asterisks indicate sulfur-containing metabolites. Presented reactions do not include all known reactions.

The *AtPGDH1* gene was previously identified as the *embryo sac development arrest 9* gene, and its mutation was embryonically lethal^35^. This gene also plays an important role in pollen development^20^. Other studies showed that the *AtPGDH1* gene is coexpressed with genes of tryptophan biosynthesis^1^, and that the activators of tryptophan biosynthesis MYB51 and MYB34 are involved in the expression of *AtPGDH1* ^1^. Thus, we think that *AtPGDH1* is responsible for the supply of serine as a substrate for tryptophan synthase (Fig. 10). In fact, tryptophan-derived metabolites such as indole acetic acid and indole glucosinolates were present at lower concentrations in *AtPGDH1*-silenced plants^1^, demonstrating that serine used as a precursor for tryptophan biosynthesis is synthesized in the phosphorylated pathway, and that the involvement of AtPGDH1 is greatest among the three isoforms. On the other hand, l-serine is also a precursor for sulfur-containing amino acids, and is converted into *O*-acetyl-l-serine and then reacts with sulfide to form l-cysteine (Fig. 10). These two reactions are catalyzed by serine acetyltransferase and *O*-acetylserine (thiol) lyase, and are finely regulated at the enzyme activity level depending on the availability of sulfur^16^. Hence, the positive and sensitive regulation of AtPGDH1 and AtPGDH3 by l-homocysteine may contribute to the balance between sulphur assimilation and tryptophan biosynthesis. l-homocysteine is converted into AdoMet via l-methionine in the *S*-adenosyl-l-methionine cycle^36, 37^. The conversion of l-homocysteine to l-methionine is associated with folate-mediated one-carbon metabolism, in which single carbon units originate from l-serine and other molecules^38^. AdoMet is converted into *S*-adenosyl-l-homocysteine and then into l-homocysteine^36^. Hence, l-homocysteine may be a signaling molecule that enhances AdoMet production by activating l-serine biosynthesis.

During photosynthesis, ribulose 1,5-bisphosphate carboxylase/oxygenase (Rubisco) is a key enzyme for carbon fixation via the Calvin cycle and forms 3-PGA by reacting with carbon dioxide (CO_2_; Fig. 10). Photorespiration is initiated by binding of oxygen to Rubisco as a substrate instead of CO_2_, resulting in glycolate formation. Accordingly, under high CO_2_ conditions where photorespiration is repressed, CO_2_ fixation is enhanced. In agreement, valine, alanine, leucine, and histidine accumulations were enhanced in wild-type *A*. *thaliana* in the presence of high CO_2_ concentrations^1^. Under these CO_2_ conditions, serine biosynthesis via the glycolate pathway is repressed, likely increasing the importance of the phosphorylated pathway. In accordance, rosette leaf development was completely repressed by high CO_2_ conditions in *AtPGDH1*-silenced *A*. *thaliana* and *AtPGDH1* expression was induced^1^, suggesting that AtPGDH1 is essential for plant adaptation to high CO_2_, and to embryogenesis and pollen development. In the present experiments, AtPGDH1 was activated by valine and alanine, which are present at increased concentrations under conditions of high CO_2_. Thus, serine biosynthesis via AtPGDH1 seems to be enhanced under high CO_2_ conditions, reflecting both transcriptional and enzymatic activation.

The mitochondrial glycolate pathway is generally considered the major source of serine in photosynthetic organs, whereas the plastid phosphorylated pathway is predominant in non-photosynthetic organs^2^. However, the relative contributions of these pathways to serine biosynthesis in various organs and at various developmental stages remain unclear. In addition, the interplay between these pathways has not been investigated. In a recent study, exogenously supplied serine induced the expression of *AtPGDH2* and *AtPGDH1* in aerial parts and roots and in aerial parts only, respectively^39^. These observations are in apparent contradiction with regulatory enzymatic feedback inhibition by serine. Moreover, the AtPGDH activating mechanisms shown herein further indicate greater regulatory intricacies of metabolic networks than those based purely on the regulation of the phosphorylated pathway by serine.

## Methods

### Multiple alignments of amino acid sequences

Amino acid sequences of PGDHs from *A*. *thaliana* (AtPGDHs), *A*. *halophytica* (AhPGDH), *M*. *tuberculosis* (MtPGDH), *R*. *norvegicus* (RnPGDH), *H*. *sapiens* (HsPGDH), and *E*. *coli* (EcPGDH) were aligned using CLUSTAL W with MEGA6 software^40, 41^. Alignments were rendered using ESPript (http://espript.ibcp.fr)^42, 43^.

Accession numbers for each amino acid sequence are as follows: AtPGDH1, NP_195146 (EMBL/GenBank/DDBJ); AtPGDH2, NP_564034 (EMBL/GenBank/DDBJ); AtPGDH3, NP_566637 (EMBL/GenBank/DDBJ); AhPGDH, BAF91727.1 (EMBL/GenBank/DDBJ); MtPGDH, 1YGY chain A (PDB); RnPGDH, CAA66374.1 (EMBL/GenBank/DDBJ); HsPGDH, NP_006614.2 (EMBL/GenBank/DDBJ) and EcPGDH 1YBA chain A (PDB).

### Reagents

Nicotine amide adenine dinucleotide (NAD) disodium salt, 3-phosphoglycerate [D-(–)-3-phosphoglyceric acid disodium salt], l-homoserine, d-homoserine, l-homocysteine, 2-oxoglutarate (α-ketoglutaric acid sodium salt), oxaloacetate, *cis*-aconitate, molecular weight standards (Gel Filtration Markers Kit for Protein Molecular Weights) for size exclusion chromatography analyses, and Murashige–Skoog (MS) vitamin solution were purchased from Sigma–Aldrich Co., Ltd. (St. Louis, MO, USA). The other amino acids, the salt mixture of MS medium, Good’s buffers, NADH (β-diphosphopyridine nucleotide disodium salt, reduced form), fumarate, malate (l-malate), citrate (citric acid monohydrate), and isocitrate [trisodium ( ± )-isocitrate n-hydrate] were purchased from Wako Pure Chemical Industries, Ltd. (Osaka, Japan).

### Plant materials

To clone *AtPGDH3* (At3g19480) cDNA, *A*. *thaliana* accession Columbia-0 (Col-0) were grown on agar-solidified half-strength MS medium containing 0.5% sucrose, 1 mM MES, and 0.1% MS vitamin solution (pH 5.8) for 3 weeks under fluorescent light/dark conditions (16-h light/8-h dark) at 22 °C.

### Preparation of recombinant enzymes

Full-length cDNA clones of *AtPGDH1* (At4g34200) and *AtPGDH2* (At1g17745) were obtained from RIKEN Bio Resource Center (accession code pda02295 and pda04481, respectively)^44, 45^. Full-length cDNA of *AtPGDH3* (At3g19480) was synthesized using a PrimeScript II High Fidelity One Step RT-PCR Kit (Takara Bio Inc., Kyoto, Japan) with total RNA from 3-week-old *A*. *thaliana* rosette leaves using the RNeasy Plant mini Kit (Qiagen) as a template. Subsequently, cDNA was amplified using the primers shown in Supplementary Table 1 and were directly inserted into pENTR-D-TOPO vectors (Life Technologies) and then sequenced.

Transit peptide sequences for chloroplast localization of *AtPGDH1*, *AtPGDH2*, and *AtPGDH3* were predicted by comparing their amino acid sequences with that of the *MtPGDH* gene. Regions that correspond with mature enzymes were amplified using the primers shown in Supplementary Table 1 and were then inserted into *Spe*I and *Not*I sites of the expression vector pPAL7 (Bio-RAD) using In-Fusion cloning with the In-Fusion HD cloning kit (Takara Bio Inc.)^46^.

Recombinant enzymes were expressed in *E*. *coli* BL21 CodonPlus (DE3)-RIL (Agilent). Pre-cultivation was performed in Luria–Bertani (LB) liquid medium containing 100 µg/mL carbenicillin and 30 µg/mL chloramphenicol at 28 °C for 12 h. Subsequently, 2% cultures were inoculated into 150 mL of LB liquid medium containing 50 µg/mL carbenicillin and 30 µg/mL chloramphenicol and cells were grown at 20 °C until OD_600_ reached 0.5. Subsequently, isopropyl β-d-thiogalactopyranoside was added (final concentration 0.5 mM) and cells were incubated for a further 12 h at 20 °C.

Tag-free recombinant proteins were prepared using a Profinity eXact Fusion-Tag System (Bio-RAD) and a Profinia Protein Purification instrument (Bio-RAD) equipped with Bio-Scale Mini Profinity eXact Cartridges (1 mL) for affinity purification. Bio-Scale Mini Bio-Gel P-6 Desalting Cartridges (10 mL) were used for desalting according to the manufacturer’s instructions. Briefly, cell pellets were obtained by centrifugation of *E*. *coli* cultures at 9,000 × *g* and were then suspended in 100 mM sodium phosphate buffer (pH 9.0) and sonicated on ice for 10 min. Crude extracts were then centrifuged for 10 min at 9,000 × *g*. Supernatants were then applied to a Profinia Protein Purification Instrument and on-column incubations were performed for 1 h at 20 °C to eliminate the affinity tag eXact Fusion-Tag from recombinant proteins. Eluted fractions were then immediately concentrated to 250 µL by ultrafiltration using an Amicon Ultra-4 Centrifugal filter Unit (MWCO 10,000; Merck-Millipore).

Domain swapping of AtPGDHs was performed as follows. Initially, regions of ASB and ACT domains from AtPGDHs were estimated using Uniport^47^ (Fig. 2). N-terminal half regions containing catalytic domains and C-terminal half regions containing ASB and ACT domains were then amplified using specific primer sets (Supplementary Table 1). Amplified N- and C-terminal fragments were then inserted into *Spe*I and *Not*I sites of the expression vector pPAL7 with combinations of AtPGDH-N1 and AtPGDH-C2 or AtPGDH-N2 and AtPGDH-C1, and homologous recombination was performed using an In-Fusion HD cloning kit^46^.

### Estimation of molecular weights of recombinant AtPGDHs

Molecular weights of wild-type and chimeric AtPGDHs in native states were estimated in size exclusion chromatography analyses (flow rate 1 mL/min) in the presence of 100 mM phosphate using AKTA explorer 10 s (for AtPGDH1, AtPGDH2 and AtPGDH3) or AKTA Start (for AtPGDH-2N/1 C and AtPGDH-1N/2 C) instruments with HiLoad 26/600 Superdex 200 preparation grade columns (GE Healthcare). Molecular weights were calculated based on standard curves of thyroglobulin (669 kDa), apoferritin (443 kDa), β-amylase (200 kDa), alcohol dehydrogenase (150 kDa), albumin (66 kDa), and carbonic anhydrase (29 kDa) in 100 mM sodium phosphate buffer (pH 9.0).

### Spectrophotometric assays of recombinant AtPGDHs

3-PGA oxidation activity was assayed in 600 µL reaction mixtures containing 0.1 M Good’s buffer (described below), 1 mM dithiothreitol, 10 mM 3-PGA, 1 mM NAD^+^, 0.1 M NaCl, and 5.0 µg of recombinant enzyme. After preincubation of reaction mixtures without 3-PGA at 25 °C for 10 min, reactions were initiated by adding 3-PGA^12, 48^. To determine substrate specificities of reduction activity, 10 mM carboxylic acid solutions (citrate, *cis*-aconitate, isocitrate, oxaloacetate, succinate, fumarate, malate, or 2-oxoglutarate) were incubated in 600 µL reaction mixtures containing 0.1 M TAPS (pH 9.0), 1 mM dithiothreitol, 0.1 mM NADH, 0.1 M NaCl, and 10 µg of recombinant enzyme. Subsequently, 3-PGA oxidation activities were determined according to increases in absorbance of NADH (340 nm), which is generated from NAD^+^ with the oxidation of 3-PGA. Conversely, carboxylic acid reduction was determined by monitoring decreases in absorbance at 340 nm using a UV-2700 spectrophotometer (Shimadzu, Kyoto, Japan). Reaction mixtures without substrates were used as negative controls, and changes in absorbance at 340 nm were monitored under the same conditions. Experiments were performed 2 times. In some cases, experimental conditions differed slightly and single representative results are presented.

Optimal pH values for 3-PGA oxidation activity were determined in the presence of 10 mM 3-PGA and 1 mM NAD^+^ at pH intervals of 0.5 between 6.0 and 11.0. Good’s buffers were used as follows: MES-NaOH for pH 6.0 and 6.5; HEPES-NaOH for pH 7.0, 7.5, and 8.0; TAPS-NaOH for pH 8.5 and 9.0; CHES-NaOH for pH 9.5 and 10.0, and CAPS-NaOH for pH 10.5 and 11.0. Data were collected from three technical replicates. The optimal pH for reduction of 2-oxoglutarate and oxaloacetate was determined using 10 mM 2-oxoglutarate or oxaloacetate and 0.1 mM NADH at pH intervals of 0.5 between 6.0 and 9.0.

To determine kinetic parameters for 3-PGA oxidation activity at pH 9.0, 3-PGA was added at 0.1, 0.2, 0.5, 0.7, 1, 2, 5, 7, and 10 mM with a fixed NAD^+^ concentration of 1 mM. In separate experiments, NAD^+^ was applied at 0.01, 0.02, 0.05, 0.07, 0.1, 0.2, 0.5, 0.7, and 1.0 mM with a fixed 3-PGA concentration of 10 mM. Initial velocities were determined from slopes of plots of NADH formation versus incubation time. To determine kinetic parameters for the reduction of 2-oxoglutarate and oxaloacetate at pH 6.0, 2-oxoglutarate and oxaloacetate were added at 1, 2, 5, 7, 10, and 20 mM with a fixed NADH concentration of 0.1 mM. Initial velocities were determined from slopes of plots of decreases in NADH versus incubation time. The molar extinction coefficient (*ε*) of NADH at 340 nm was 6.2 × 10^3^. Kinetic parameters [apparent Michaelis constants (*K*_m_^app^) and apparent maximum velocities (*V*_max_^app^)] were calculated by fitting specific activities (*v*) to Michaelis-Menten equations under various concentrations of substrate (S)^49^ as follows:

$$v={V}_{max}{\rm{S}}/({K}_{{\rm{m}}}+{\rm{S}})$$

where *V*_max_ and *K*_m_ represent the maximal velocities and the Michaelis constants, respectively. Data fitting was performed using the Enzyme kinetics module in SigmaPlot (Systat Software, San Jose, CA).

To determine whether chloride ions activate 3-PGA oxidation activity, specific activities were determined at 10 mM 3-PGA and 1 mM NAD^+^ in the presence of 0, 10, 20, 50, 70, 100, 200, and 500 mM NaCl or KCl. Data were collected from three technical replicates.

Sensitivity to various amino acids was tested using the aforementioned method with slight modifications. Initially, the effects of amino acids were determined at 10 mM by calculating specific activities of the enzyme. Subsequently, specific activities were determined at 10 mM 3-PGA and 1 mM NAD^+^ in the presence of various concentrations of amino acids as follows: for AtPGDH1, AtPGDH2, and AtPGDH3, l-homoserine, l-alanine, l-valine, l-cysteine, and d-cysteine were added at 0, 0.1, 0.2, 0.5, 0.7, 1, 2, 5, 7, 10, 20, and 50 mM; for AtPGDH1, l-methionine was added at 0, 0.01, 0.02, 0.05, 0.07, 0.1, 0.2, 0.5, 0.7, 1, 2, 5, 7, 10, 20, and 50 mM; for AtPGDH2, l-methionine was added at 0, 0.1, 0.2, 0.5, 0.7, 1, 2, 5, 7, 10, 20, and 50 mM; for AtPGDH3, l-methionine was added at 0, 0.1, 0.2, 0.5, 0.7, 1, 1.5, 2, 2.5, 3, 3.5, 4, 4.5, 5, 7, 10, 20, and 50 mM; for AtPGDH1 and AtPGDH3, l-serine was added at 0, 0.1, 0.2, 0.5, 0.7, 1, 2, 5, 7, 10, 20, and 50 mM; for AtPGDH2, l-serine was added at 0, 0.1, 0.2, 0.5, 0.7, 1, 2, 5, 7, and 10 mM; for AtPGDH1, l-homocysteine was added at 0, 0.001, 0.002, 0.005, 0.007, 0.01, 0.015, 0.02, 0.025, 0.03, 0.035, 0.04, 0.045, 0.05, 0.055, 0.06, 0.07, 0.1, 0.2, 0.5, 0.7, and 1 mM; for AtPGDH2, l-homocysteine was added at 0, 0.001, 0.002, 0.005, 0.007, 0.01, 0.02, 0.05, 0.07, 0.1, 0.2, 0.5, 0.7, and 1 mM; for AtPGDH3 l-homocysteine was added at 0, 0.001, 0.002, 0.005, 0.007, 0.01, 0.02, 0.03, 0.04, 0.05, 0.06, 0.07, 0.08, 0.09, 0.1, 0.2, 0.5, 0.7, and 1 mM. To determine the effects of amino acids on AtPGDH-N2/C1 and AtPGDH-N1/C2 activities, ranges of amino acid concentrations were set as follows: l-serine, l-valine, l-homoserine, and l-methionine, 0, 0.1, 0.2, 0.5, 0.7, 1, 2, 5, 7, 10, 20, and 50 mM; l-homocysteine, 0, 0.001, 0.002, 0.005, 0.007, 0.01, 0.02, 0.05, 0.07, 0.1, 0.2, 0.5, 0.7, and 1 mM; l-alanine, 0, 0.1, 0.2, 0.5, 0.7, 1, 1.5, 2, 2.5, 3, 3.5, 4, 4.5, 5, 7, 10, 20, and 50 mM. To determine combined effects of activators in the presence of l-serine, ranges of amino acid concentrations were set as follows: l-methionine, l-valine, l-homoserine, and l-alanine, 0, 0.1, 0.2, 0.5, 0.7, 1, 2, 5, 7, 10, 20, and 50 mM; l-homocysteine, 0.01, 0.02, 0.05, 0.07, 0.1, 0.2, 0.5, 0.7, 1, 2, and 5 mM.

### Non-linear regression with Hill equations

Cooperativities and half maximal effective concentrations (EC_50_) of effectors were determined by fitting percentage relative activities (the specific activity at 0 mM was set at 100) of effectors (E) at various effector concentrations (C) to the sigmoidal E_max_ model with baseline response (the Hill equation)^24^ as follows:

$${\rm{E}}={{\rm{E}}}_{0}+{{\rm{E}}}_{{\rm{\max }}}{{\rm{C}}}^{{{\rm{n}}}_{{\rm{H}}}}/({{{\rm{EC}}}_{50}}^{{{\rm{n}}}_{{\rm{H}}}}+{{\rm{C}}}^{{{\rm{n}}}_{{\rm{H}}}})$$

where E_0_ represents the relative activity in the absence of effector (baseline), E_max_ represents the relative activity at the maximal effective concentration (inhibition or activation) of the effector, n_h_ is the Hill coefficient, and EC_50_ is half maximal effective concentration of the effector at half E_max_.

The parameters E_0_, E_max_, n_H_, and EC_50_ were estimated using three or six data sets for percentage relative activities (E) under various effector concentrations (C). The data were then fitted to the sigmoidal E_max_ model with baseline response using the nonlinear least squares method (Levenberg-Marquardt algorithm) without weight functions using SigmaPlot. In the tables, EC_50_ and n_h_ values are shown as estimated values ± standard errors only when *p* values for n_h_ are smaller than 0.015.

## Electronic supplementary material

Supplementary Information
