# Supplementary material for: Novel regulatory mechanism of serine biosynthesis associated with 3-phosphoglycerate dehydrogenase in Arabidopsis thaliana
Source: Sci Rep. 2017 Jun 14;7:3533. doi: 10.1038/s41598-017-03807-5 (PMC5471267; doi:10.1038/s41598-017-03807-5)
Supplement: Supplementary file 1 — Supplementary Information [file 41598_2017_3807_MOESM1_ESM.pdf]

Supplementary Information

Title: Novel regulatory mechanism of serine biosynthesis associated with 3-phosphoglycerate dehydrogenase in *Arabidopsis thaliana*

Authors: Eiji Okamura and Masami Yokota Hirai*

Author affiliation: RIKEN Center for Sustainable Resource Science, 1-7-22 Suehiro-cho, Tsurumi-ku, Yokohama, Kanagawa 230-0045, Japan

Contact information: Masami Yokota Hirai

RIKEN Center for Sustainable Resource Science, 1-7-22 Suehiro-cho, Tsurumi-ku, Yokohama, Kanagawa 230-0045, Japan

Telephone number: +81-45-503-9491

E-mail address: masami.hirai@riken.jp

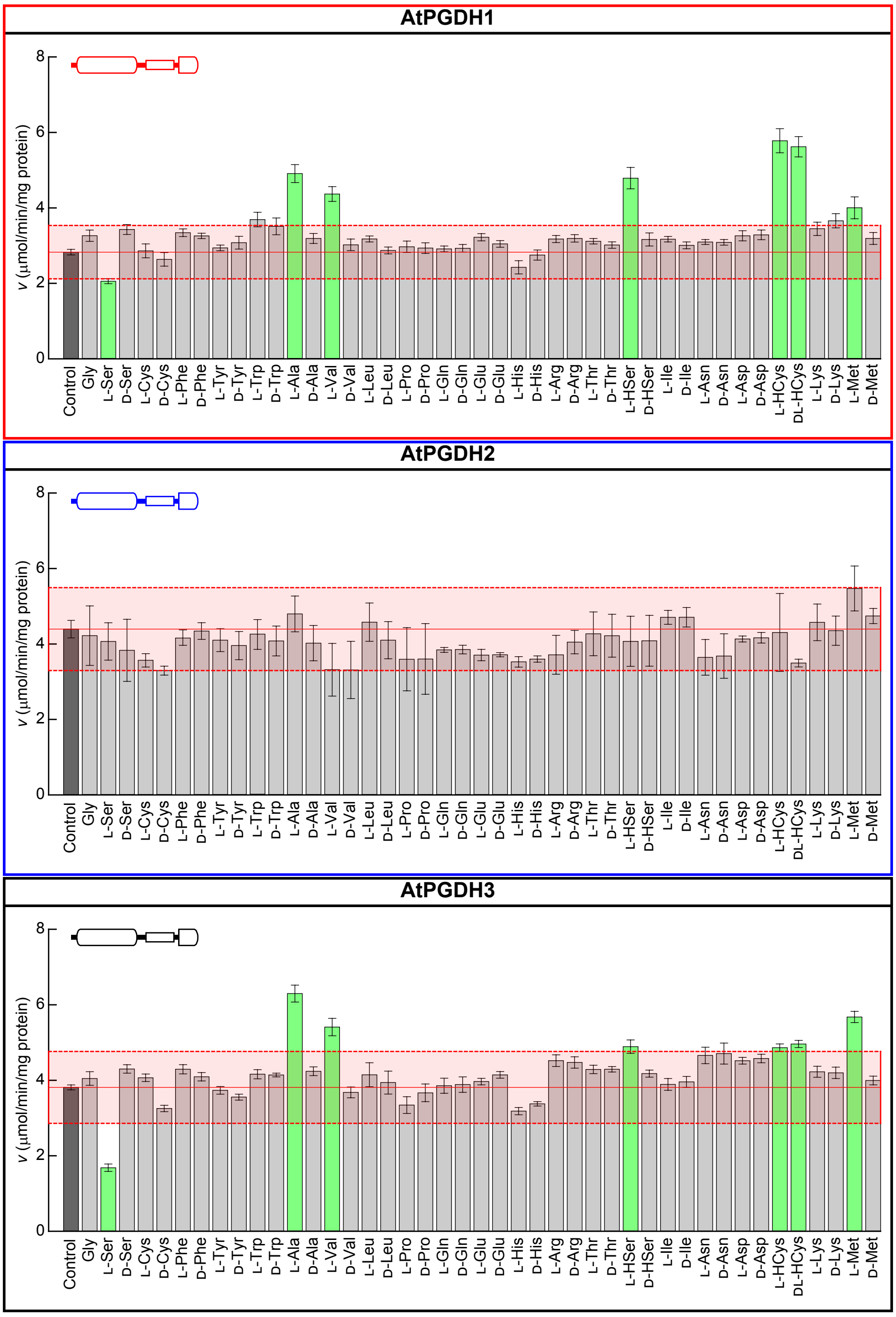
AtPGDH1
AtPGDH2
AtPGDH3
v (µmol/min/mg protein)
0
2
4
6
8
Control
Gly
L-Ser
D-Ser
L-Cys
D-Cys
L-Phe
D-Phe
L-Tyr
D-Tyr
L-Trp
D-Trp
L-Ala
D-Ala
L-Val
D-Val
L-Leu
D-Leu
L-Pro
D-Pro
L-Gln
D-Gln
L-Glu
D-Glu
L-His
D-His
L-Arg
D-Arg
L-Thr
D-Thr
L-HSer
D-HSer
L-Ile
D-Ile
L-Asn
D-Asn
L-Asp
D-Asp
L-HCys
DL-HCys
L-Lys
D-Lys
L-Met
D-Met

Supplementary Figure 1 | Effects of amino acids on 3-PGA oxidation activity of AtPGDHs. The vertical axes represent specific activity. Green bars indicate statistically significant differences ($p < 0.001$) by >25% compared with the specific activity in the absence of amino acid (control). Red shaded area indicates change by <25% compared with control. The mean and standard error of two biological replicates with three technical replicates ($n = 6$) are shown.

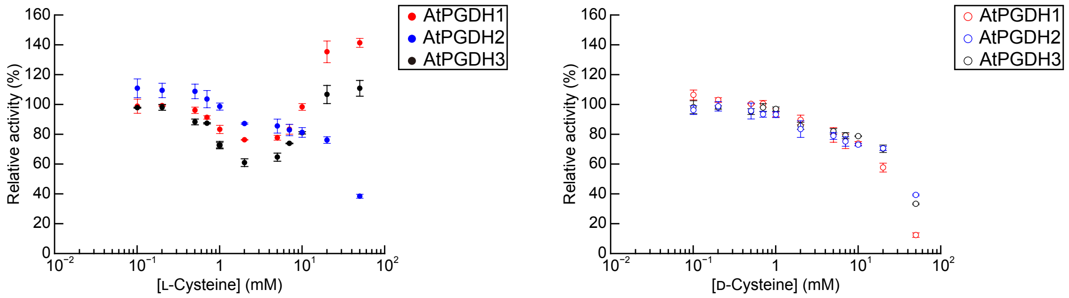

AtPGDH1
AtPGDH2
AtPGDH3
Relative activity (%)
[L-Cysteine] (mM)
AtPGDH1
AtPGDH2
AtPGDH3
Relative activity (%)
[D-Cysteine] (mM)

Supplementary Figure 2 | Dose response of 3-PGA oxidation activity of AtPGDHs to cysteine. The specific activities of AtPGDHs at different concentrations of L- and D-cysteine were measured and are shown as relative activities to those measured in the absence of L- and D-cysteine. The mean and standard error of three technical replicates are shown.

Supplementary Table 1 | Primers used in this study

AtPGDH1-SpeI	AAGCTCTTCAAAGCTTTGACTAGTTCTAAGCCTACGATTCTCGTGGCG	for mature AtPGDH1enzyme
AtPGDH1-NotI	CGGGCTTATGCGGCCCTAGAGCTTGAGGAAAACGAACTC	for mature AtPGDH1enzyme
AtPGDH2-SpeI	AAGCTCTTCAAAGCTTTGACTAGTCCAAAGCCGAGGATCCTCGTCACC	for mature AtPGDH2 enzyme
AtPGDH2-NotI	CGGGCTTATGCGGCCTTATAGTTTAAGAAACACAAACT	for mature AtPGDH2 enzyme
AtPGDH3-SpeI	AAGCTCTTCAAAGCTTTGACTAGTGGAAAACCGACGATTCTCGTA	for mature AtPGDH3 enzyme
AtPGDH3-NotI	CGGGCTTATGCGGCCCTATAGTTTGAGGAAAACAAACTC	for mature AtPGDH3 enzyme
AtPGDH1-N	*CACACCGCTTCCTCCGGCCACCAGTTGCAC*	for AtPGDH-N1/C2
AtPGDH1-C	**GGAGGTAAAGGAGTA***AAAAACGCGAAAATCACATACGCCTCAGCA*	for AtPGDH-N2/C1
AtPGDH2-N	**TACTCCTTTACCTCCAGATGCTAACTGCAC**	for AtPGDH-N2/C1
AtPGDH2-C	**GGAGGAAGCGGTGTG***CAGTCCATTAGGGTGGTCTATCGG*	for AtPGDH-N1/C2

Primer sequences are written in the 5′ to 3′ direction. Underlined, double-underlined, italicized and bold-faced sequences denote pPAL7 vector sequence, coding sequences of AtPGDHs, sequences of AtPGDH1 and AtPGDH2, respectively.
